# Employing temporal self-similarity across the entire time domain in computed tomography reconstruction

**DOI:** 10.1098/rsta.2014.0389

**Published:** 2015-06-13

**Authors:** D. Kazantsev, G. Van Eyndhoven, W. R. B. Lionheart, P. J. Withers, K. J. Dobson, S. A. McDonald, R. Atwood, P. D. Lee

**Affiliations:** 1 Manchester X-ray Imaging Facility, School of Materials, University of Manchester, Manchester M13 9PL, UK; 2 Research Complex at Harwell, Didcot, Oxfordshire OX11 0FA, UK; 3 iMinds-Vision Lab, University of Antwerp, 2610 Wilrijk, Belgium; 4 School of Mathematics, University of Manchester, Alan Turing Building, Manchester M13 9PL, UK; 5 Department of Earth and Environmental Sciences, Ludwig Maximilian University, Munich, Germany; 6 Diamond Light Source, Harwell Science and Innovation Campus, Didcot OX11 0DE, UK

**Keywords:** iterative reconstruction, spatial–temporal regularization, time lapse tomography, non-local means, structural prior

## Abstract

There are many cases where one needs to limit the X-ray dose, or the number of projections, or both, for high frame rate (fast) imaging. Normally, it improves temporal resolution but reduces the spatial resolution of the reconstructed data. Fortunately, the redundancy of information in the temporal domain can be employed to improve spatial resolution. In this paper, we propose a novel regularizer for iterative reconstruction of time-lapse computed tomography. The non-local penalty term is driven by the available prior information and employs all available temporal data to improve the spatial resolution of each individual time frame. A high-resolution prior image from the same or a different imaging modality is used to enhance edges which remain stationary throughout the acquisition time while dynamic features tend to be regularized spatially. Effective computational performance together with robust improvement in spatial and temporal resolution makes the proposed method a competitive tool to state-of-the-art techniques.

## 1. Introduction

In many situations in X-ray tomographic imaging, it is not possible to collect enough data for good-quality reconstructions using conventional filtered backprojection techniques [1]. Examples can be found in medical imaging, where the accumulated dose must be kept to a minimum and in the imaging of quickly evolving events, where the time per projection or the number of projections must be severely reduced in order to capture the temporal dynamics of the scanned sample. In such cases, iterative techniques can provide better reconstructions [2].

When dealing with iterative image reconstruction there is a strong need for regularization techniques which impose *a priori* information on the desired solution [2,3]. The nature of this information can be different, for example, some local or non-local (NL) neighbour correlations can be encouraged [4]. In some cases, additional information can be extracted not only from the spatial domain but also from the temporal space [5,6]. Sometimes, it is possible to augment the main reconstruction dataset with supplementary information using the same or a different imaging modality [7,8]. Normally, the other modality dataset will have different image characteristics, such as intensity, resolution, geometry and noise variation. This can restrict the ‘direct’ embedding of the prior information into the reconstruction process [8].

Previously, there have been successful attempts to improve spatial resolution in time-lapse tomography using prior information [9–12]. This supplementary information is normally obtained before the time-lapse experiment (e.g. a pre-scan at high resolution) and regarded as the reference image. For example, in [10], the assumption about the prior image is provided without the explicit use of regularization which leads to improvement in resolution. The use of a high-resolution image to regularize the main dataset is already a well-established approach, and one of the most common approaches in this area is prior image constrained compressed sensing (PICCS) [9], which employs a high-quality prior image in the sparse regularization framework to improve spatial resolution.

In [11], supplementary information is provided to improve an NL regularization strategy. NL image regularization [13], which is based on successful NL denoising methods [14], has been commonly applied to image reconstruction problems [15–17] and also to time-lapse reconstruction [11,12,18].

In this paper, we present a novel multi-modal NL regularization technique which uses a supplementary dataset to drive a spatio-temporal (ST) regularization process for time-lapse tomography. We use a prior image of higher resolution that can be from the same or a different imaging modality, which distinguishes our method from the previously proposed mono-modal algorithms [9–12]. Additionally, the proposed algorithm employs all the available temporal information (not just adjacent time frames as in [18]) which greatly improves the signal-to-noise ratio (SNR) of reconstructions. The prior image is used to select the most structurally valuable neighbours for temporal regularization (a pre-classification strategy), which also leads to improved spatial resolution and substantially accelerates numerical performance.

In common with [12], we aim to minimize the computational complexity and achieve a sufficient trade-off for ST resolution while using NL regularizers. While the method in [12] sacrifices temporal resolution to improve spatial resolution, we aim to restore the desirable balance by introducing a constraint which restricts regularization across dissimilar time frames.

The proposed method is compared to the state-of-the-art PICCS regularization technique and shows much more promising results when the given prior image is not ideal (noisy and/or partially uncorrelated with the imaged dataset).

It should be noted that in the current state our method is well suited for a specific class of video denoising or time-lapse reconstruction problems. Specifically, our technique has the potential to significantly enhance edges which remain stationary throughout the acquisition experiment while dynamic features tend to be regularized spatially. In material science, our method is well suited to problems such a fluid flow through rigid porous structures such as rocks [12], solid oxide fuel cells [19] and bioscaffolds [20].

## 2. Method

### (a) Parallel beam time-lapse tomography

A discrete representation of the stationary attenuation coefficients to be reconstructed can be written as a system of linear equations
2.1
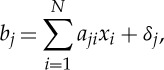

where *b*_*j*_,*j*=1,…,*M* is the measured projection data (sinogram) and *M* is the total number of projections, *x*_*i*_, *i*=1,…,*N* is the discrete distribution of attenuation coefficients to be reconstructed (*N* is the total number of image elements) and *δ*_*j*_ is the noise component in the measurements *b*_*j*_. Weights *a*_*ji*_∈[0,1] (contribution of element *i* to the value detected in the bin *j*) form the sparse system matrix 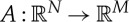.

Let us consider a problem in which part of the image changes over time and the other part remains effectively stationary. Writing equation (2.1) in a matrix–vector form and adding the temporal dimension gives
2.2
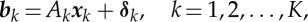

where *K* is a total number of three-dimensional time frames. Similar to the algorithm in [12] we use all available time frames.

The explicit (direct) solution for (2.2) can be written as 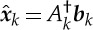 with a pseudo-inverse 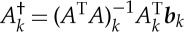. This direct inversion (if practically possible) is highly sensitive to noise due to amplification of high-frequency components: 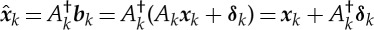. In our case, the system of equations (2.2) is severely underdetermined (*M*≪*N*) and the system matrix *A* is ill-conditioned. To find an approximate solution 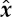 from the undersampled noisy measurements, one can choose regularized iterative techniques instead of direct approaches [2,3].

In this paper, we aim at reconstructing iteratively the set of images ***x***_*k*_ while adding a novel ST regularization penalty.

### (b) Regularized time-lapse iterative reconstruction algorithm

Define 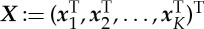 as the vector containing all images of the time-lapse series and similarly define the measured projections vector as 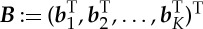. Therefore, the system of equations to solve is ***B***=***AX***, where the block diagonal matrix ***A*** is given as follows:
2.3
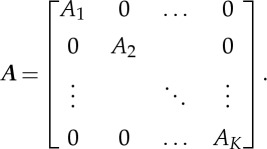



The traditional approach to solve a linear system of equations, such as (2.1), is to find the best fit 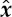 to the exact ***x*** using the least-squares (LS) approximation [21]. In other words, one would like to minimize the ℓ_2_ norm between the forward projections and the measured projection data:
2.4
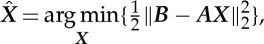

where 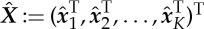. The optimization problem (2.4) is quadratic and can be solved using gradient-based techniques, such as the conjugate gradient least-squares (CGLS) algorithm [21]. To turn (2.4) into a well-posed problem, one has to *regularize* the solution ***X*** by adding a *penalty* term *R*(***X***), resulting in the following regularized problem:
2.5
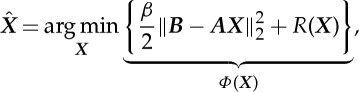

where *β* is a regularization parameter which represents the trade-off between the data fidelity and the regularization term.

The gradient of the cost function *Φ*(***X***) can be calculated as follows:
2.6
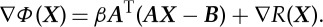

Rather than using direct minimization approaches (e.g. gradient descent) to solve problem (2.5) one can use splitting techniques [22]. The idea is to split the data fidelity and regularization terms using proximity operators. This approach leads to simpler stackable optimization problems, such as forward–backward splitting (FBS) or Bregman-type methods [15]. Applied to our minimization problem (2.5), the estimate 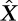 can be computed using the following two-step FBS algorithm:
2.7
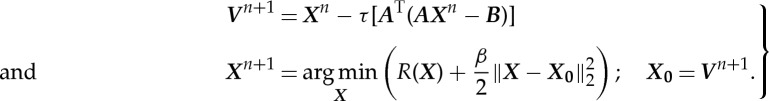

In the above algorithm, one can see that the first step solves the unregularized LS problem, and the second is the data term dependent image denoising step [15]. To accelerate convergence of (2.7), we will replace the gradient descent (GD) minimization (first step) with the CGLS algorithm [21]. Although CGLS converges faster than GD, the overall convergence proof for (2.7) method does not hold anymore [22]; however, in practice this combination provides successful results [18]. The main focus of our interest here is the nature of the penalty term *R*(***X***).

### (c) Non-local means-based spatio-temporal regularization

The discrete representation of the ST regularization term is based on NL gradient [15,16] and given by
2.8
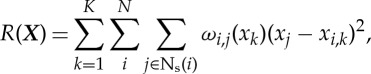

where the *search* domain N_s_ is restricted to the volumetric neighbourhood size of *N*_search_×*N*_search_×*K* with the number of neighbours equal to *N*^2^_search_*K*. Note that the volumetric search area N_s_ includes all time-frames *K*. Non-negative and symmetric weights *ω*_*i*,*j*_ are calculated as follows:
2.9
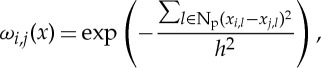

where N_p_ is a quadratic *similarity* patch size of *N*_sim_×*N*_sim_ and parameter *h* corresponds to the noise level in ***x***.

The Euler–Lagrange equation of the second minimization problem in (2.7) with the penalty term (2.8) is as follows:
2.10
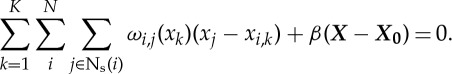

With the weight term fixed, the Euler–Lagrange equation (2.10) is linear and GD-based schemes can be used to find the solution. Here we used the fixed point minimization scheme to solve (2.10) efficiently [23]
2.11
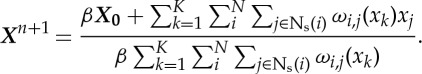



As can be seen from the ST regularizer (2.8), there is no special treatment for *x*_*i*,*t*_;*t*=[1,2,…,*K*]∖{*k*} elements which are dissimilar to *x*_*i*,*k*_. When the intensity of *x*_*i*,*t*_ is different from the intensity of *x*_*i*,*k*_ element there is a probability that the information in *t* frame is quite different from the current time frame *k*. Therefore, if regularization is unconstrained for *t* frame it can potentially lead to over-smoothing of dynamic (or dissimilar) features [12]. Similar to the method introduced in [17], we constrain the regularization across potentially dissimilar time frames with the following rule:
2.12
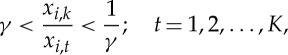

where *γ* is a constant. For every *i*th element in time frame *k*, we check that the *i*th element in different time frame *t* is similar in terms of intensity. If elements are dissimilar ((2.12) is not fulfilled) the temporal frame *t* is not considered for regularization within the search space N_*s*_(*i*). During our experiments, we found that condition (2.12) and the choice of *γ* is critical to avoid smoothing of dynamic features.

Although the proposed ST penalty term can handle random noise in reconstructed images much better than just a spatial penalty, the current implementation is computationally infeasible. In the next section, we will show how additional information can be embedded into (2.8) to improve spatial resolution and significantly reduce computational time.

### (d) Embedding structural information into spatio-temporal regularization

Let *z*_*i*_,*i*=1,…,*N* be a supplementary dataset, then the structural information can be extracted from ***z*** in the following way. The following similarity measure is calculated as:
2.13
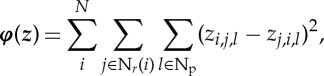

where N_*r*_ is a quadratic similarity patch size of *N*_search_×*N*_search_. The vector ***φ***, calculated for every *z*_*i*_, provides distribution of similarity values within the window N_*r*_. Smaller values in ***φ*** demonstrate higher similarity to *z*_*i*_ and by sorting values from low to high, one can choose *n*_0_ of the most similar to *z*_*i*_ elements:
2.14
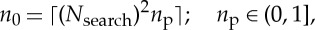

where *n*_p_ is an empirically chosen parameter which controls the number of *j*th elements in N_*r*_ taken to build a structural set.

Let us define a structural set *S*_*z*_(*i*,*n*_0_,*N*_*r*_) which consists of *n*_0_ most similar to *z*_*i*_ elements within the quadratic window N_*r*_. The set *S*_*z*_(*i*,*n*_0_,N_*r*_) is created according to the selection rule (2.14). If the supplementary image ***z*** has an improved resolution over ***x***_*k*_ and images have structural similarity (at least partially), then one can use the set *S*_*z*_(*i*,*n*_0_,N_*r*_) to drive the regularization process. The main aim of structural set *S*_*z*_(*i*,*n*_0_,N_*r*_) is to reduce dimensionality of the volumetric search space N_*s*_(*i*) in (2.8). The modified set 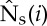 has the same spacial dimensions as *N*_s_(*i*), but the number of neighbours for regularization process is reduced to *n*_0_*K*. One can see that 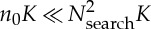 when *n*_p_≪1 in (2.14).

This approach is similar to the one which is used for multi-modal image reconstruction [8]; however since it is NL, it is more stable to noise than just using local voxel absolute differences [12]. This means that the proposed technique is a much more robust way of extracting additional information from a prior image which also can be degraded with noise or image artefacts.

### (e) Pseudocode for the proposed non-local spatio-temporal algorithm

Here we present a pseudocode for time-lapse tomographic reconstruction using the proposed structurally driven NLST penalty (2.8).


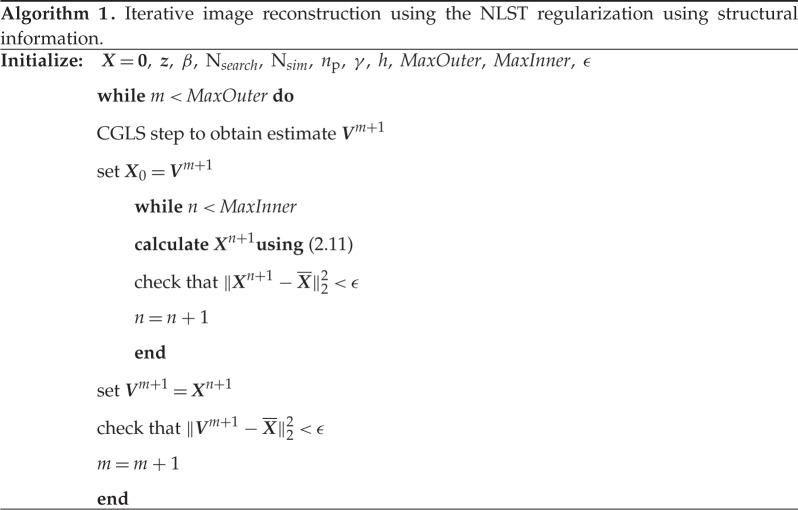


## 3. Numerical experiments

In this section, two different numerical experiments are performed, which demonstrate the improvement of the proposed NLST technique over a state-of-the-art PICCS method [17]. The aim of the PICCS method is the same as the method proposed and involves the integration of a prior image into the reconstruction process to improve ST resolution. The optimization problem for PICCS using the total variation (TV) penalty [24] and a prior image ***z*** is given as follows:
3.1
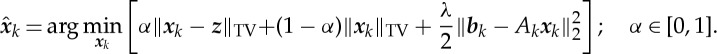

We perform PICCS optimization with respect to each time frame ***x***_*k*_. The main goal of (3.1) is to find the best approximation to each time frame ***x***_*k*_ when ***z*** is available and the trade-off between ***x***_*k*_ and ***z*** is controlled by the parameter *α*. Note that PICCS is not using all available temporal information as the NLST method but is based solely on the prior image ***z*** and the current time frame ***x***_*k*_. We optimized (3.1) using FBS splitting where the LS term was solved independently with CGLS and the PICCS minimization sub-problem was performed with the GD method using the time-step parameter *τ* (table 1).

**Table 1.** Parameters for the image reconstruction experiment (figure 5).

parameter	method	value	description
*MaxOuter*	NLST	11	outer iterations (CGLS) number in algorithm 1
*MaxInner*	NLST	1	inner iterations number in algorithm 1
*N*_search_	NLST	11	the size of the searching window
*N*_sim_	NLST	3	the size of the similarity window
*n*_p_	NLST	0.05	the number of *n*_0_ neighbours (2.14)
*β*	NLST	2.6	regularization parameter (2.11)
*h*	NLST	0.15	noise-dependent threshold (2.9)
*γ*	NLST	0.9	parameter in (2.12)
*MaxOuter*	PICCS	12	outer iterations (CGLS) number
*MaxInner*	PICCS	25	inner GD iterations number
λ	PICCS	0.01	regularization parameter (3.1)
*α*	PICCS	0.4	trade-off parameter (3.1)
*τ*	PICCS	0.001	time-step parameter for GD
*ϵ*	NLST and PICCS	1×10^−5^	an iteration tolerance constant

To avoid storing the large sparse matrix *A*, we used on-the-fly forward and backward projection operations of the GPU accelerated modules from the ASTRA toolbox [25]. C-OMP implementation using a Matlab wrapper of the proposed NLST algorithm (2.8) is freely available [26].

To quantify our results, we use two measures. The first measure is the root mean square error (RMSE):
3.2
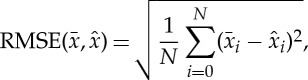

where 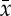 is the exact image and 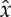 is a reconstructed image. And the second is the structural similarity index (SSIM) [27] which is given as
3.3
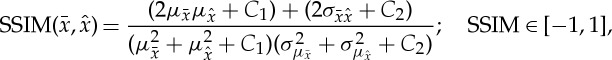

where *μ* and *σ* are the mean intensity and standard deviation of image block, respectively (we used an 8×8 quadratic patch). 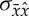 denotes the cross-correlation and *C*_1,2_ are small constants to avoid singularity [27]. SSIM is a more advanced quality measure than RMSE (3.2), as it considers image degradation as a visually perceived change in structural information. The SSIM value equals 1 if images are identical.

We optimized thoroughly all the reconstruction parameters (see §3a) and the optimal parameters are given in table 1. The videos with reconstructed data (experimental and real) are available in the electronic supplementary material.

### (a) Image reconstruction of modelled data

**Figure 1.**
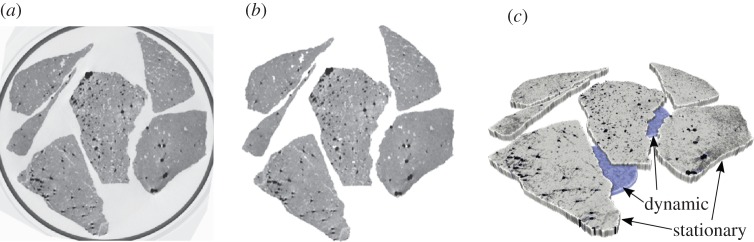
(*a*) Reconstruction of the porous granitic gravel sample from 2000 projections using the Feldkamp algorithm; (*b*) realistic rock phantom created from the image; (*c*) rendered three-dimensional phantom (*x*,*y*+time) where stationary and dynamic ROIs are shown.

Similar to [12], a synthetic dynamically changing phantom for time-lapse tomographic image reconstruction was created as follows. First, a high-quality reconstruction based on an X-ray projection dataset collected for a rock sample (porous granitic gravel), which was acquired on a Nikon XTH 225 ST cone beam scanner at the Manchester X-ray facility, was reconstructed with the Feldkamp algorithm. This reconstruction is displayed in figure 1*a*. Based on this reconstruction, the rock region was extracted and all other attenuation values were set to zero, resulting in the image displayed in figure 1*b*. Next, fluid flow was simulated in the void space region, where the time points at which fluids enters a certain voxel were randomly generated by applying a global thresholding operation on a two-dimensional Perlin noise image [28]. The stationary and dynamic regions of interest (ROIs) are shown in figure 1*c*.

In this experiment, we simulated two cases, namely cases where 45 and 25 projections were taken per time frame (30 time frames in total) resulting in 1350 and 750 projections, respectively. Projections were collected using the golden ratio (GR) firing-order technique [29]. The GR scanning approach is used to obtain projections in a non-sequential order. The basic idea is to adapt the angular sequence of projections so that any subsets of chronologically contiguous projections contain sufficient information for reconstruction. This technique is well suited to iterative reconstruction methods, because one can divide the scan into an arbitrary number of subscans which are normally sampled below the Nyquist rate. Each projection was generated with a strip kernel [1] and a higher resolution version of the phantom, i.e. on an 800×800 isotropic pixel grid. Poisson distributed noise was applied to the projection data, assuming an incoming beam intensity of 30 000 (photon count). Reconstructions were calculated on a 300×300 isotropic pixel grid and with a linear projection model [1], thus avoiding the ‘inverse crime’ of generating the data with the same model as the model that is used for calculating the reconstruction. In total, 30 different time frames were reconstructed by subdividing the simulated projection data into 30 distinct subsets of 45 and 25 projections each.

**Figure 2.**
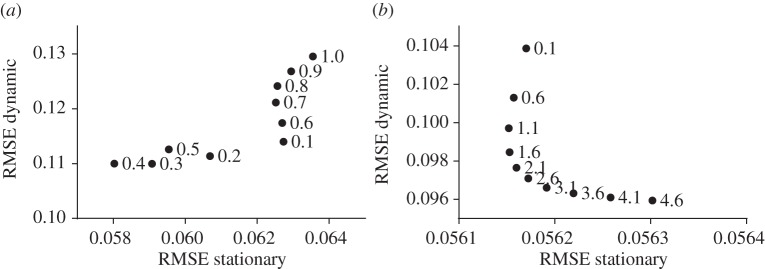
Optimization procedure to find the optimal values of (*a*) *α* selection for the PICCS method (3.1) and (*b*) *β* selection for the NLST method (2.11). The optimization was performed with respect to RMSE values in stationary and dynamic ROIs of the phantom (figure 1*c*).

For a fair comparison of the CGLS–PICCS and CGLS–NLST methods, we initially optimized the parameters (see parameters in table 1). In figure 2, we present the result of the final optimization procedure for *α* of PICCS and *β* of the NLST method. Other parameters previously chosen to be optimal (or nearly optimal) are fixed as shown in table 1.

**Figure 3.**
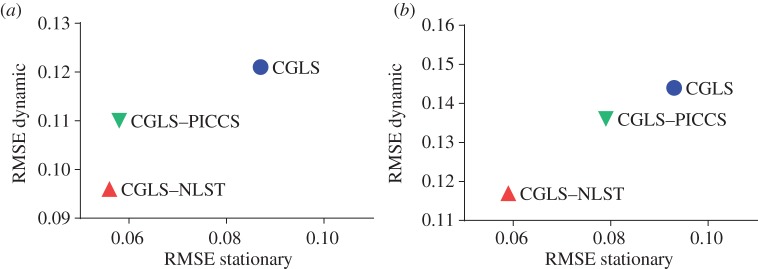
RMSE values for the whole dataset 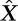 reconstructed with different methods from (*a*) 45 and (*b*) 25 projections per time frame *k*. The proposed regularization method outperforms the CGLS–PICCS and CGLS methods.

In figure 3, we show the obtained RMSE values for the CGLS, CGLS–PICCS and CGLS–NLST methods for cases when 45 and 25 projections are used to reconstruct each time frame. One can see that the proposed CGLS–NLST method outperforms CGLS–PICCS in both cases. Notably, for the case reconstructed from 25 projections per time frame the difference in RMSE values between NLST and PICCS becomes more apparent (figure 3*b*). Those results demonstrate that the proposed method is more robust in dealing with under-sampled noisy projection data.

**Figure 4.**
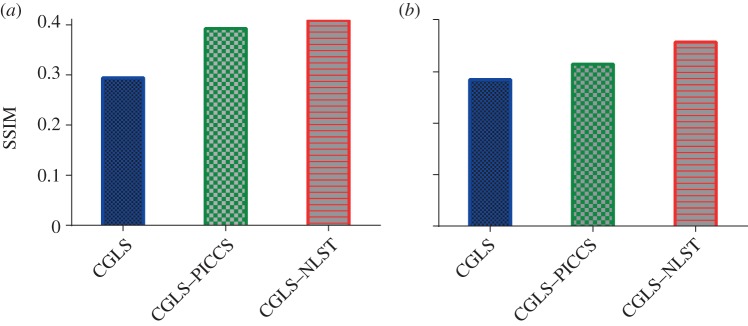
SSIM values for the whole dataset 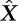 reconstructed with different methods from (*a*) 45 and (*b*) 25 projections per time frame *k*. The proposed method slightly outperforms the CGLS–PICCS method for 45 projections reconstruction case and more significantly for 25 projections.

**Figure 5.**
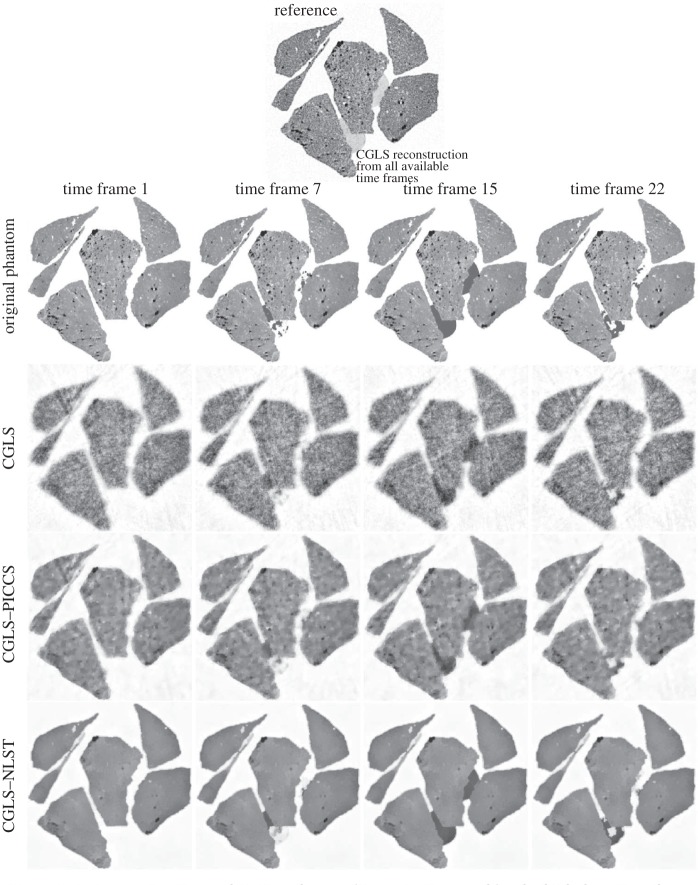
Two-dimensional reconstructions of 30 time frames (45 projections each), of which four time frames are shown. The presented images were reconstructed using the CGLS method (10 iterations), CGLS–PICCS and CGLS–NLST methods. The reference image (top) is reconstructed with the CGLS method (15 iterations) from 1350 noisy projections and contains averaged dynamic ROI. The images reconstructed with the proposed method demonstrate high spatial and temporal resolution and low level of noise.

**Figure 6.**
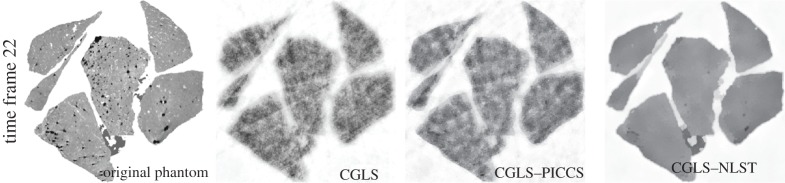
Two-dimensional reconstructions of 30 time frames (25 projections each), of which one time frame (*k*=22) is shown. For reconstruction with CGLS–PICCS and CGLS–NLST the same reference image used as in figure 5 (top). The CGLS–NLST method strongly outperforms the CGLS–PICCS method here.

The SSIM values were calculated for the reconstructed datasets and shown in figure 4. The time frames *k*=1,7,15,22 from the whole reconstructed dataset 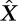 for 45 projections are shown in figure 5. One time frame *k*=22 is shown in figure 6 where reconstruction from 25 projection angles is performed.

For reconstructions with the CGLS–PICCS and CGLS–NLST methods (figures 5 and 6), we used the reference image which was reconstructed with the CGLS method from 1350 noisy dynamically changing projections (figure 5 (top)). Note that the reference image is noisy and dynamic resolution is lost through time averaging in the reconstruction process. In figures 5 and 6, one can see that the CGLS–PICCS method is able to improve spatial resolution while using the reference image; however the noise level is high. The proposed CGLS–NLST method delivers significant improvement in spatial and temporal resolution and SNR.

Reconstruction from 25 projections per time frame (figure 6) demonstrates that the proposed method strongly outperforms CGLS–PICCS for under-sampled noisy projection data. Quantitatively, there is also a significant difference in values between the two methods (figures 3 and 4).

**Figure 7.**
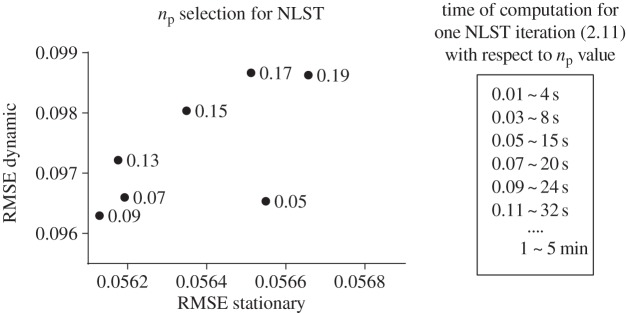
The effect of the *n*_p_ parameter on the accuracy of reconstruction and the computation time. The optimal value is *n*_p_=0.09 and the computation speed is less than 30 s for one fixed point iteration (2.11). The data parameters are 300×300×30 pixels and 4 Intel CPU cores i5 (2.5 GHz) were used.

The choice of *n*_p_ parameter in (2.14) is important since it reduces the search space (less time for computation) and also drives the regularization process based on the reference image which results in improved resolution. In figure 7, we demonstrate that the optimal value for *n*_p_ is around 0.09 and the computation time with this value is less than 30 s for one fixed point iteration (2.11). This is more than 10 times faster than if we take the whole searching space *n*_p_=1,*n*_0_=(*N*_search_)^2^.

## 4. Real data tomographic reconstruction

Here we present numerical results for a real tomographic reconstruction problem of dynamically evolving objects. Tomographic inversion in this case is severely under-determined and projection data are contaminated by random noise and artefacts (rings and streaks).

The tomographic experiment (experiment ee10500-1) was performed at I12 JEEP beamline facility of the Diamond Light Source synchrotron (Harwell, UK). The flow of potassium iodide solution through a bead-pack was imaged by suspending the flow outlet tube over the centre of a rotating 15 mm diameter sample holder thereby allowing a controlled supply of fluid into the sample (bimodal glass beads 1:1 by mass of 0.5 mm and 1 mm diameter). The column of beads was rotating at approximately 3 Hz. The sample was illuminated with direct monochromatic X-rays of 53 keV energy. A Vision Research Miro 310M camera was used to acquire the images using a 200–900 μs exposure and a projection acquisition rate of 1080 frames per second. Prior to flow, a high-resolution ‘dry’ scan was obtained with 1800 projections in 180°. During the flow a continuous sequence of over 18 000 dynamically evolving ‘wet’ projections were acquired with 180 projections over 180°.

**Figure 8.**
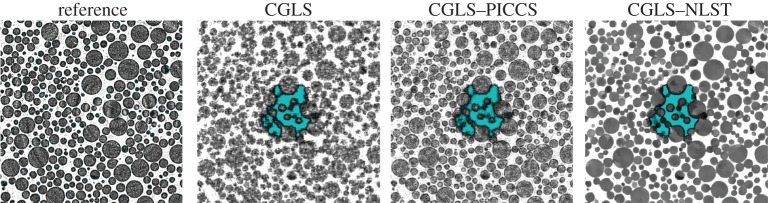
Magnified ROI of the glass beads dataset (one horizontal slice from one of the 30 volumetric time frames) showing the ingress of the liquid. The ‘dry’ reference image is reconstructed with 20 CGLS iterations and used in the CGLS–PICCS and CGLS–NLST algorithms. One can see that the CGLS–NLST method gives the best spatial resolution and sharpest contrast between the liquid and glass particles.

**Figure 9.**
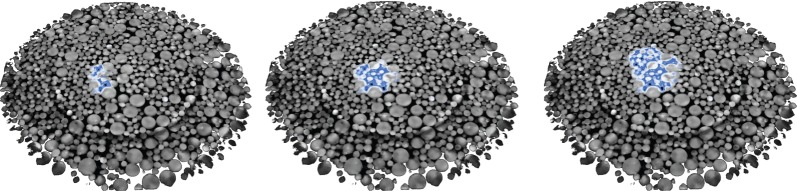
Rendered time-lapse sequence of the liquid ingress into the glass beads is shown. The volumes (only 50 slices are shown) were reconstructed using the CGLS–NLST method from 90 projections per time frame (time frames *k*=1,7,15 were taken).

We down-sampled the resulting data to 500 projections for the ‘dry’ scan and the dynamically evolving data (‘wet’) to 90 projections per time frame. The size of each two-dimensional XY slice is 1024×1024 pixels and due to parallel geometry each slice can be reconstructed independently. The ‘dry’ scan was reconstructed iteratively (20 iterations) with CGLS (figure 8) and used as a prior image for the CLGS–PICCS and CGLS–NLST methods. The reference image has sharp contrast (all sizes of glass particles are visible), but some level of noise and reconstruction artefacts are present. We reconstructed 30 dynamically changing volumes and one slice of one of the time frames, where liquid is present, is shown for the CGLS, CGLS–PICCS and CGLS–NLST methods (figure 8) and show how the dynamic information within the datasets can be rendered for subsequent qualitative and quantitative analysis (figure 9). The CGLS reconstruction has poorer resolution and higher noise level. The CGLS–PICCS successfully embeds the prior information into the reconstruction resulting in higher resolution, but overall the reconstruction is noisy. The proposed CGLS–NLST method produces denoised image with the sharpest contrast and distinctly outlined liquid front (central ROI). The sharp contrast between liquid and glass particles will significantly alleviate the post-processing step.

Here we comment on the process of choosing the optimal parameters for the compared methods for real data reconstruction. Although the CGLS–PICCS method has a smaller number of controlled parameters (table 1), it has been much more difficult (compared to the CGLS–NLST method) to find the optimal (visually pleasing) parameters for CGLS–PICCS. In contrast to the CGLS–NLST method, we used exactly the same set of parameters as in table 1 (only *β* was chosen differently); however for the CGLS–PICCS method we were optimizing for the λ and *α* parameters. If the prior image is not ideal (as in our case), it is more difficult with CGLS–PICCS to find the best trade-off between the noise level present in the data and the prior image as well as to avoid blurring of dynamically changing features. We conclude that the proposed CGLS–NLST method is robust to noise in the prior images, is aware of dynamic features (different from the prior image) present in the data and is easy to use.

## 5. Discussion

Exploiting all the available time frames in ST regularization is a challenging task and a good balance is required between spatial and temporal resolution. For the proposed method, we assume that some features are fixed in time and can be spatially enhanced by the temporal correlation. Because of this requirement, not every time-lapse tomographic dataset is suitable for the proposed method. The approach is thus limited to cases where some features are aligned in time (otherwise there is no benefit of using this approach) and the prior image is registered to the main dataset. Although the computation time on multiple CPUs (OMP realization in C language [26]) is significantly reduced with the proposed approach (which makes it feasible even for large datasets), a GPU implementation has the potential to accelerate this method even further with a massive thread parallelization.

The reference image can be obtained by scanning the object for a longer period of time prior to the dynamic experiment. If the prior image is not available, one can use the reconstructed image (as a reference) from all collected projection data as is shown in the modelled numerical experiment (see §3a). If there is no direct way to obtain a good estimate to constrain regularization, one should consider methods similar to [12].

## 6. Conclusion

In this paper, we presented results of a novel ST regularization technique which is based on NL methods for image denoising. Our method is generalized to employ all available temporal information and the supplementary data. By employing the temporal correlation of repetitively imaged objects and available prior information, it is possible to achieve a higher spatial resolution, SNR and speed of computation in comparison to the state-of-the-art reconstruction algorithms.

In the current state, this method has the potential for dynamic tomographic applications where some parts of the imaged object are fixed and others are varying over time. The flexibility of the proposed regularizing penalty and ease of computer implementation make it transferable across a wide range of imaging applications.

## Supplementary Material

Electronic Supplementary Material - S1

## Supplementary Material

Electronic Supplementary Material - S3

## Supplementary Material

Electronic Supplementary Material - S3
