# Supplementary figures and images for: Employing temporal self-similarity across the entire time domain in computed tomography reconstruction
Source: Philos Trans A Math Phys Eng Sci. 2015 Jun 13;373(2043):20140389. doi: 10.1098/rsta.2014.0389 (PMC4424485; doi:10.1098/rsta.2014.0389)

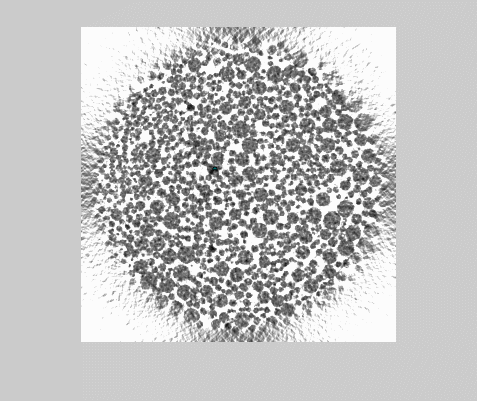

Supplement: Electronic Supplementary Material - S1 [file rsta20140389supp1.gif]

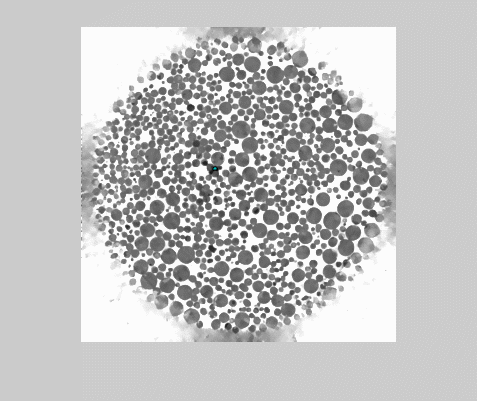

Supplement: Electronic Supplementary Material - S3 [file rsta20140389supp2.gif]

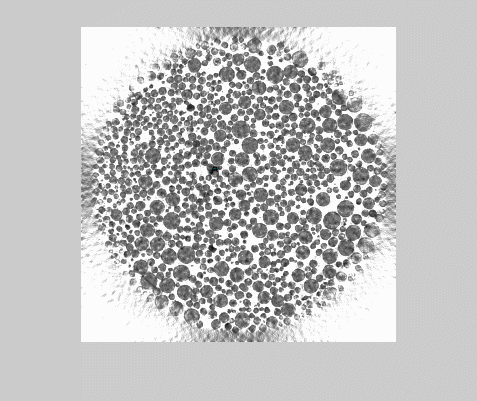

Supplement: Electronic Supplementary Material - S3 [file rsta20140389supp3.gif]
